# Supplementary material for: Bis(Disulfide)-Bridged Somatostatin-14 Analogs and Their [111In]In-Radioligands: Synthesis and Preclinical Profile
Source: Int J Mol Sci. 2024 Feb 5;25(3):1921. doi: 10.3390/ijms25031921 (PMC10856354; doi:10.3390/ijms25031921)
Supplement: Supplementary file 1 [file ijms-25-01921-s001.zip › ijms-2822231-SI.pdf]

International Journal of *Molecular Sciences*

MDPI

Article

Supplementary Materials: Bis(Disulfide)-Bridged Somatostatin-14 Analogs and Their [^{111}In]In-Radioligands: Synthesis and Preclinical Profile

Aikaterini Tatsi [1,2], Theodosia Maina [1,*], Beatrice Waser [3], Eric P. Krenning [4], Marion de Jong [5 †], Jean-Claude Reubi [3], Paul Cordopatis [2 †] and Berthold A. Nock [1]

ESI-MS and HPLC Results of Newly Synthesized AT5S and AT6S

The ESI-MS spectra and representative HPLC chromatograms for the new bicyclic 6/12-mer and 8/12-mer analogs AT5S and AT6, are shown in the following Figures S1-S4, confirming the formation and the high purity of the products.

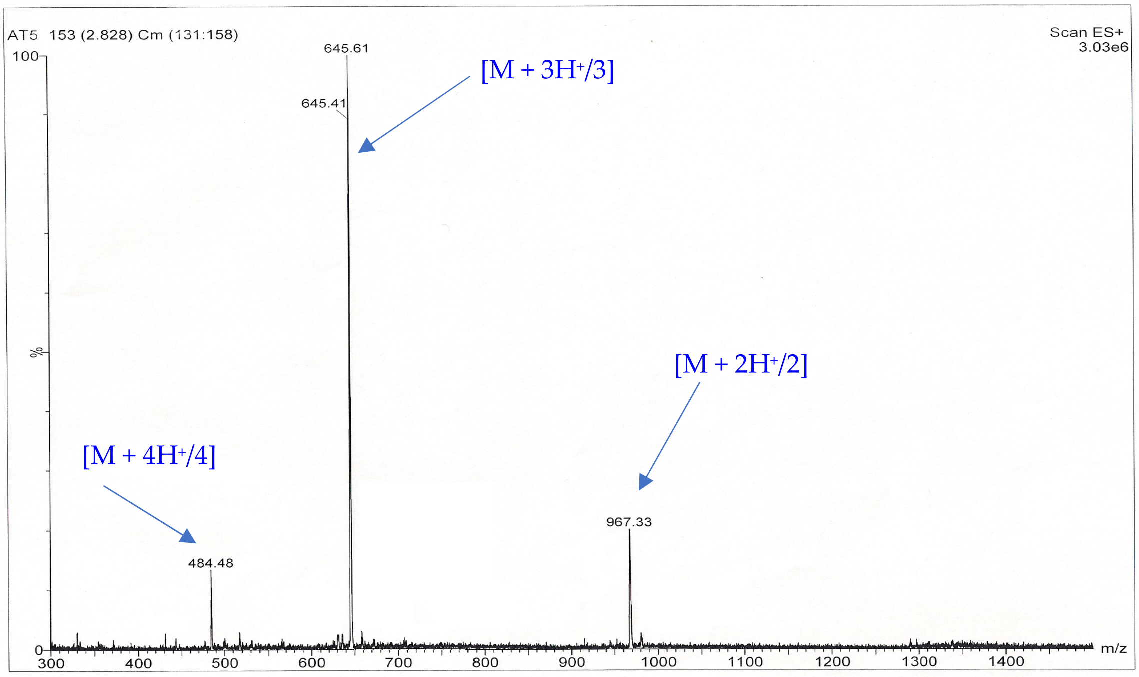

Figure S1. ESI-MS spectra confirming the formation of bicyclic 6/12-member-ring AT5S (DOTA-Ala^{1}-Gly^{2}-c[Cys^{3}-Lys^{4}-Asn^{5}-c[Cys^{6}-Phe^{7}-DTrp^{8}-Lys^{9}-Thr^{10}-Cys^{11}]-Thr^{12}-Ser^{13}-Cys^{14}]; DOTA, 1,4,7,10-tetraazacyclododecane-1,4,7,10-tetraacetic acid); Fragment ion peaks found by ESI-MS on a Micromass-Platform LC instrument (Waters Micromass Technologies, Milford, MA, USA): [M+2H^{+}/2]: 967.3, [M+3H^{+}/3]: 645.6, and [M+4H^{+}/4]: 484.5.

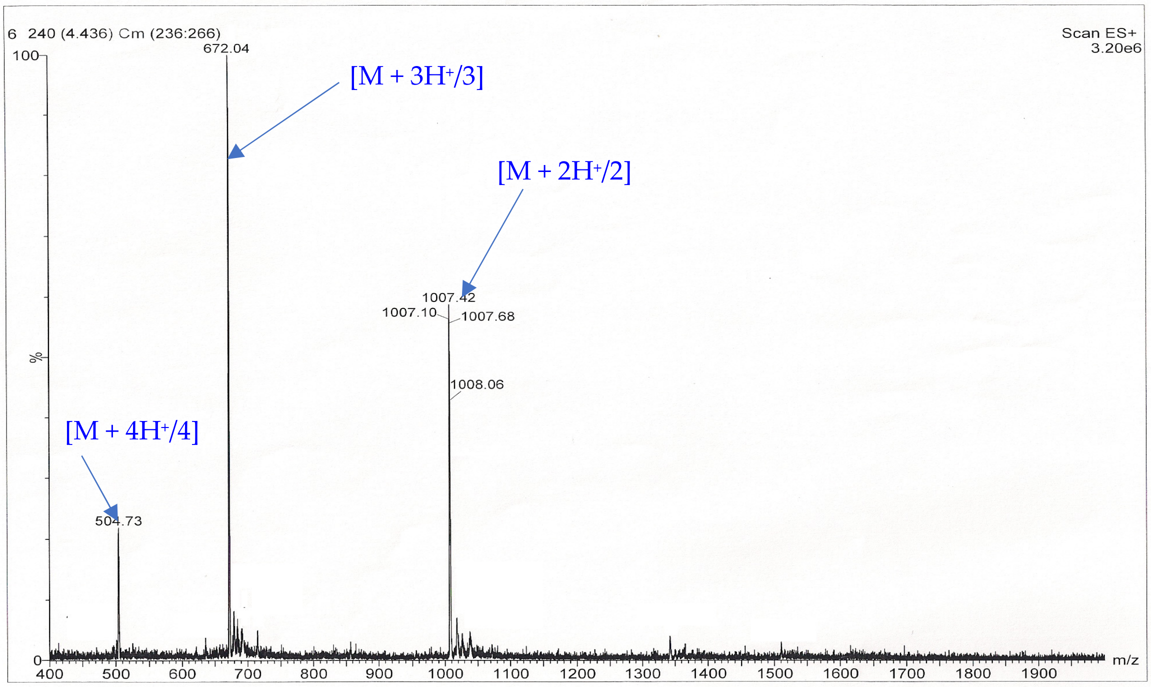

Figure S2. ESI-MS spectra confirming the formation of bicyclic 8/12-member-ring AT6S (DOTA-Ala[1]-Gly[2]-c[Cys[3]-Lys[4]-c[Cys[5]-Phe[6]-Phe[7]-DTrp[8]-Lys[9]-Thr[10]-Phe[11]-Cys[12]]-Ser[13]-Cys[14]]); Fragment ion peaks found by ESI-MS on a Micromass-Platform LC instrument (Waters Micromass Technologies, Milford, MA, USA): [M+2H+/2]: 1007.4, [M+3H+/3]: 672.4, and [M+4H+/4]: 504.7.

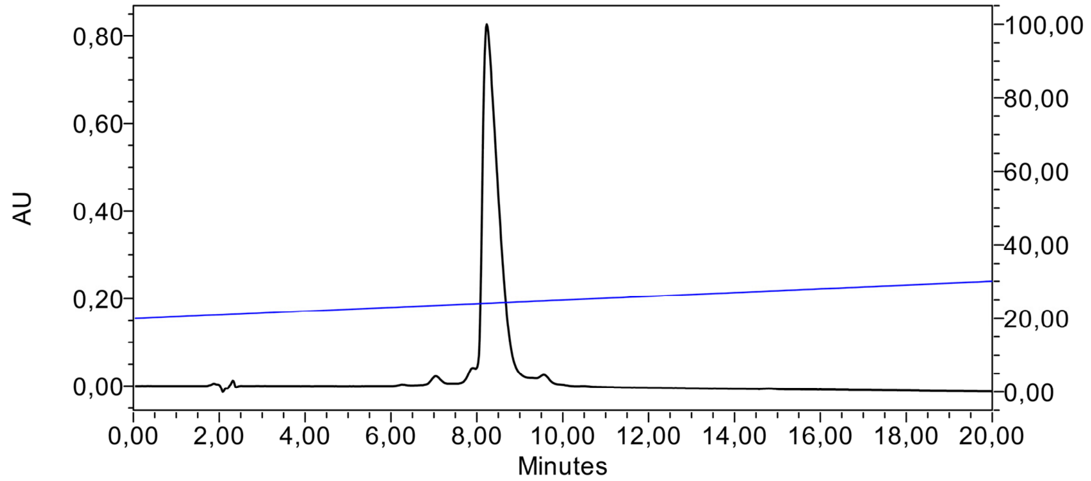

(**a**)

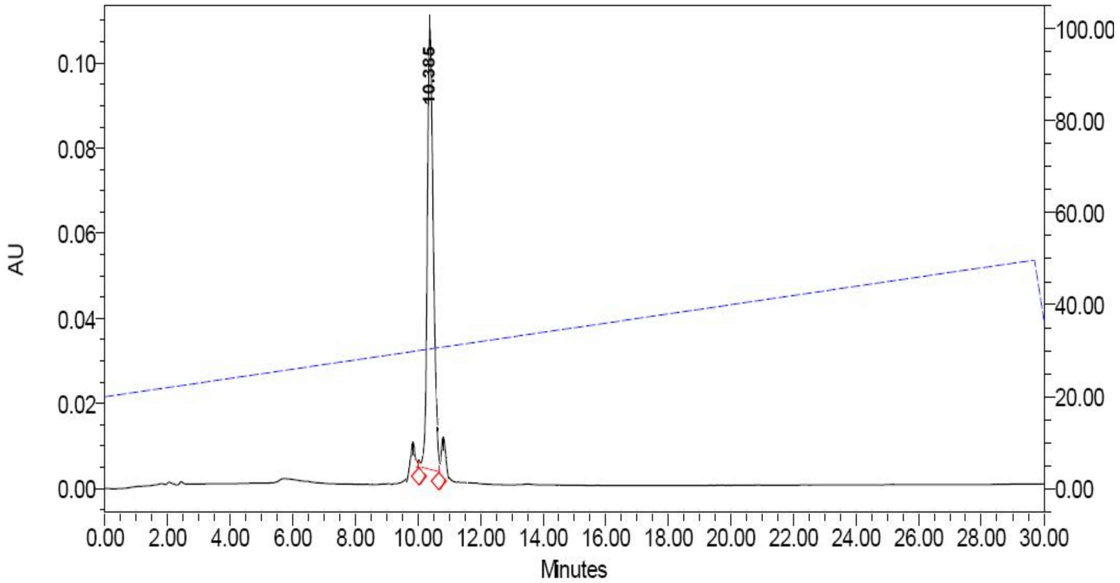

(**b**)

Figure S3. HPLC UV trace at 220 nm of the analysis of AT5S; (**a**) System 1: RP-HPLC on an XBridge™ Shield RP18 cartridge column (5 µm, 4.6 mm × 150 mm; Waters, Eschborn, Germany) eluted at a 1 mL/min flow rate with a linear gradient from 80%A/20%B to 60%A/40%B in 40 min, t_R = 8.2 min; and (**b**) System 2: : RP-HPLC on a Symmetry C18 analytical column (3.5 µm, 4.6 mm × 75 mm; Waters, Micromass Technologies, Milford, MA, USA) eluted at a 1 mL/min flow rate with a linear gradient from 90%A/10%B to 50%A/50%B in 20 min, whereby A = 0.1% aqueous TFA, B = MeCN; t_R = 10.34 min; ≥ 94%.

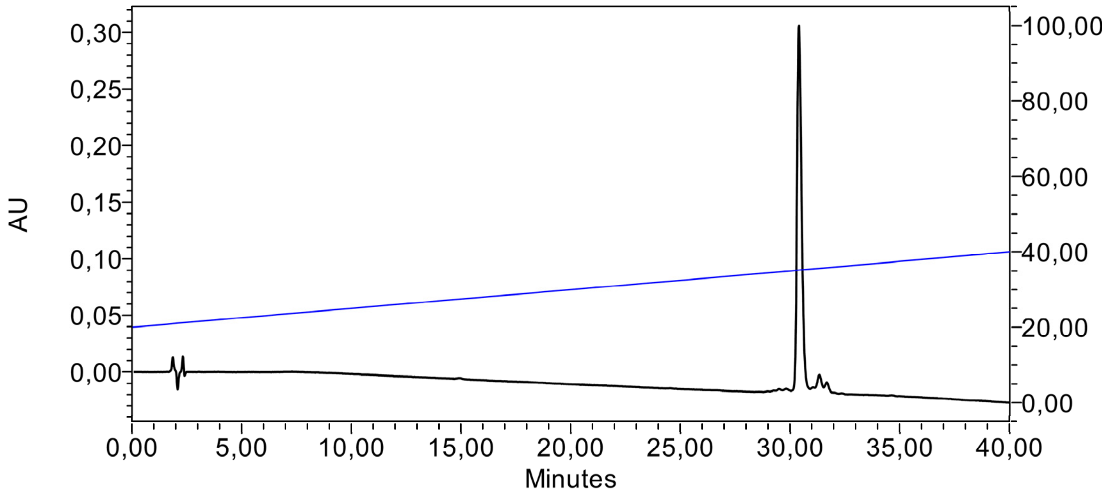

(**a**)

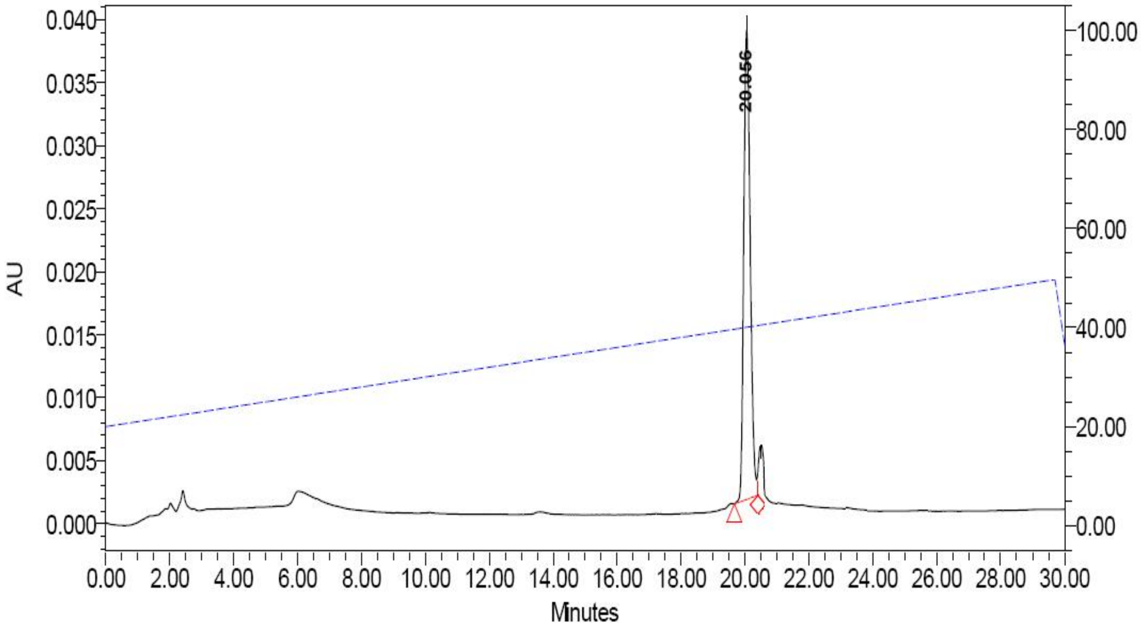

(**b**)

Figure S4. HPLC UV trace at 220 nm of the analysis of AT6S; (**a**) System 1: RP-HPLC on an XBridge™ Shield RP18 cartridge column (5 µm, 4.6 mm × 150 mm; Waters, Eschborn, Germany) eluted at a 1 mL/min flow rate with a linear gradient from 80%A/20%B to 60%A/40%B in 40 min, t_R = 30.4 min; and (**b**) System 2: : RP-HPLC on a Symmetry C18 analytical column (3.5 µm, 4.6 mm × 75 mm; Waters, Micromass Technologies, Milford, MA, USA) eluted at a 1 mL/min flow rate with a linear gradient from 90%A/10%B to 50%A/50%B in 20 min, whereby A = 0.1% aqueous TFA, B = MeCN; t_R = 20.06 min; ≥ 93%.
